# Digital Breast Tomosynthesis Complements Two-Dimensional Synthetic Mammography for Secondary Examination of Breast Cancer

**DOI:** 10.5334/jbsr.2457

**Published:** 2021-11-05

**Authors:** Eiji Nakajima, Hiroko Tsunoda, Mariko Ookura, Kanako Ban, Yuko Kawaguchi, Mami Inagaki, Norihiko Ikeda, Kinya Furukawa, Takashi Ishikawa

**Affiliations:** 1 Tokyo Medical University Ibaraki Medical Center, JP; 2 St. Luke’s International Hospital, JP; 3 St. Luke’s MediLocus, JP; 4 Tokyo Health Service Association, JP; 5 Inagaki breast clinic, JP; 6 Tokyo Medical University Hospital, JP; 7 Tokyo Medical University, JP

**Keywords:** Breast cancer, mammography, digital breast tomosynthesis, two-dimensional synthetic mammography, and full-field digital mammography

## Abstract

**Objective:**

To compare the performance of two-dimensional synthetic mammography (SM) combined with digital breast tomosynthesis (DBT) (SM/DBT) and full-field digital mammography (FFDM) including women with DBT (FFDM/DBT) undergoing secondary examination for breast cancer.

**Material and Methods:**

Out of 186 breasts, including 52 with breast cancers; FFDM/DBT and SM/DBT findings were interpreted by four expert clinicians. Radiation doses of FFDM, SM/DBT, and FFDM/DBT were determined. Inter-rater reliabilities were analyzed between readers and between FFDM/DBT and SM/DBT by Cohen’s Kappa coefficients. Diagnostic accuracy was compared between SM/DBT and FFDM/DBT by Fisher’s exact tests. Two representative cancer cases were examined for differences in the interpretation between FFDM and SM.

**Results:**

A higher radiation dose was required in FFDM/DBT than in SM/DBT (median: 1.50 mGy vs. 2.95 mGy). Inter-rater reliabilities were similar between both readers and modalities. Both sensitivity and specificity were equivalent in FFDM/DBT and SM/DBT (*p* = 0.874–1.00). Compared with FFDM, SM did not clearly show abnormalities with subtle margins in the two representative cancer cases.

**Conclusion:**

SM/DBT had a similar performance to FFDM/DBT in detecting breast abnormalities but requires less radiation. DBT complements SM to improve accuracy to a level equivalent to that of FFDM. Taken together, SM/DBT may be a good substitute for FFDM/DBT for the secondary examination of breast cancer.

## Introduction

Previous studies have shown favorable results regarding full-field digital mammography (FFDM). combined with digital breast tomosynthesis (DBT) (FFDM/DBT) for breast cancer screening [1,2,3,4,5,6,7,8]. DBT provides an added diagnostic advantage of breast imaging from different angles with less breast tissue superimposition [9]. As the radiation dose for DBT is similar to or slightly higher than the dose required for FFDM, FFDM/DBT requires approximately double the radiation dose required by FFDM alone [10]. Two-dimensional synthetic mammography (SM) images can be reconstructed from DBT images. SM combined with DBT (SM/DBT) reduces the radiation dose required due to the elimination of FFDM, while maintaining a high sensitivity [11,12,13,14,15]. DBT is currently used for secondary examination of symptomatic women or women recalled after initial screening in several countries [16,17]. In this study, we compared the performance of FFDM/DBT and SM/DBT for secondary examination of breast cancer. We hypothesized that FFDM/DBT and SM/DBT would display similar sensitivity, specificity, and accuracy, while SM/DBT would require less radiation than FFDM/DBT.

## Materials and Methods

### Study subjects

This retrospective study received ethics approval from the relevant Institutional Review Board and informed consent was obtained from all subjects. A series of 93 *nationality* women who were undergoing their first outpatient visit to the Breast Cancer Clinic of the hospital in April 2015 were enrolled. They had visited the hospital upon referral regarding their symptoms or recalled after screening. FFDM, SM, and DBT images were acquired from 186 breasts of 93 women. Subjects were excluded if they had breast implants or if images were only available for one breast. Of the 93 subjects, 47 had breast cancer, and 5 had bilateral breast cancers. Therefore, out of the 186 breasts, 52 (28.0%) breasts had cancer confirmed by the histopathological analyses of biopsied or resected specimens. Nine breasts had ductal carcinoma *in situ* (DCIS), and 43 breasts had invasive ductal carcinoma (IDC). Among the 134 breasts without breast cancer, 92 had no abnormalities, 14 had benign lesions confirmed by mammography and ultrasound, leaving 28 that had pathological benign lesions confirmed by biopsy or cytology (***Table 1***).

**Table 1:** Characteristics of the breasts analyzed.


AGE	BREAST COMPOSITION	CANCER (N = 52)	HISTOLOGICALLY BENIGN (N = 28)

31–82 years (median: 45 years)	A: 1 /B: 22 /C: 57 /D: 13 Dense breast 70 (75.3%)	DCIS 9 (17.3%) IDC 43 (82.7%)	Fibroadenoma 7 Intraductal papilloma 2 Apocrine metaplasia 1 Hemangioma 1 Phyllodes tumor 1 Negative 16


186 breasts in 93 *nationality* women. A. fatty, B. scattered fibroglandular densities, C. heterogeneously dense, D. extremely dense, DCIS, ductal carcinoma *in situ*; IDC, invasive ductal carcinoma; Negative, no indication of cancer.

### Image acquisition of FFDM, DBT, and SM constructed from DBT

FFDM and DBT images were acquired by a 70 and 100 micron pixel size, (Selenia Dimensions version 1.7; Hologic, Marlborough, MA). Breasts were compressed in a conventional manner, with an x-ray tube moving along a 15-degree arc. DBT images were reconstructed into 1-mm slices and a high-performance graphic processing unit (GPU) processor converted the 15 projection images by tomosynthesis into 1-mm thick images. The acquisition unit was set at the radiation dose used for a series of low-dose projection images. In addition to DBT, SM images were constructed from the tomosynthesis slices, i.e.: The high-performance GPU processor analyzed the slices and created a two-dimensional synthetic image (Selenia Dimensions version 1.7 plus C-View; Hologic, Marlborough, MA). For the three imaging methods (FFDM, SM, and DBT), four images (mediolateral oblique (MLO) and craniocaudal (CC) images of each breast) were yielded from each subject.

### Interpretation

**Figure 1:**
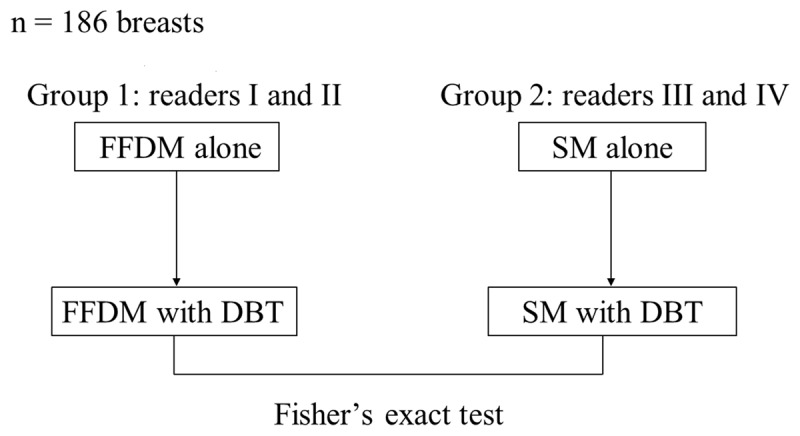
Two sequential modes were full-field digital mammography (FFDM) followed by FFDM/digital breast tomosynthesis (DBT) (Group 1), and two-dimensional synthetic mammography (SM) followed by SM/DBT (Group 2). FFDM/DBT and SM/DBT were compared using Fisher’s exact test.

Each image was independently interpreted by four medical doctors (readers I, II, III, and IV) with reading certification from the *country* Central Organization on Quality Assurance of Breast Cancer Screening (license level: sensitivity >90% and specificity >92%). Breast composition and abnormalities in the images were classified according to the Breast Imaging Reporting and Data System (BI-RADS), Atlas 5^th^ Edition. The classification of breast composition consisted of (A) fatty, (B) scattered fibroglandular densities, (C) heterogeneously dense, and (D) extremely dense. A dense composition was defined as including (C) heterogeneously and (D) extremely dense. In the present study, out of the 186 breasts examined: (Subject’s median age, 45 years; range, 31–82 years), 70 (75.3%) breasts were classified as dense (including 61.3% heterogeneously and 14.0% extremely dense) (***Table 1***). Abnormalities included masses, calcifications, focal asymmetric densities, and distortions. For each image classification, no malignancy required both the readers to interpret the breast as without any lesions or with obvious benign lesions. A probability of malignancy required that one of the readers interpreted the breast as probably benign, suspicion of malignancy, or highly suggestive of malignancy. FFDM, DBT, and SM were interpreted in two sequential modes, which were FFDM followed by FFDM/DBT (Group 1) and SM followed by SM/DBT (Group 2). The four readers were divided as follows: readers I and II interpreted Group 1 images, and readers III and IV interpreted Group 2 images (***Figure 1***).

### Evaluation of FFDM and SM images

To demonstrate the limitations of SM, the conspicuity of those lesions which were not seen on SM (70 micron pixel size), but were visible on FFDM (100 micron pixel size), were evaluated. The comparison was performed side-by-side for FFDM and SM.

### Statistical and imaging analysis

Inter-rater reliability was analyzed with Cohen’s Kappa coefficients. In Group 1, the coefficient between readers I and II was analyzed for FFDM/DBT. Similarly, in Group 2, the coefficient between readers III and IV was analyzed for SM/DBT. The coefficient was analyzed between FFDM/DBT by Group 1 and SM/DBT by Group 2 (***Figure 1***). Cohen’s Kappa coefficients were classified as follows: (a) Slight agreement, less than 0.20; (b) fair agreement, 0.20 to 0.40; (c) moderate agreement, 0.40 to 0.60; (d) substantial agreement, 0.60 to 0.80; (e) almost perfect or perfect agreement, 0.80 to 1.00 [18]. Regarding the interpretation methods (FFDM/DBT and SM/DBT), the sensitivity in 52 breasts with cancer, the specificity in 134 breasts without cancer and the accuracy in 186 breasts were calculated for the classification of no/probable malignancy. Differences in the calculated values between methods were analyzed using Fisher’s exact tests for qualitative variables. The statistical tests were two-sided and a *p*-value <0.05 was considered to indicate a statistically significant difference. All analyses were performed using the statistical software package SPSS version 25 (SPSS Corp., Chicago, IL).

## Results

Regarding the median radiation dose per one image, FFDM required 1.47 mGy and DBT required 1.50 mGy. The radiation dose in SM/DBT was the same as the dose in DBT because SM was reconstructed from DBT. FFDM/DBT required a median radiation dose of 2.95 mGy, two times larger than the dose in FFDM and SM/DBT (***Table 2***).

**Table 2:** Radiation dose for image acquisition.


		RADIATION DOSE (MGY)	MEDIAN

FFDM	MLO	0.69–6.29	

	CC	0.70–6.18	1.47

DBT (=SM/DBT)	MLO	0.92–5.43	

	CC	0.93–5.03	1.50

FFDM/DBT	MLO	1.64–11.72	

	CC	1.63–11.21	2.95


FFDM, full-field digital mammography; DBT, digital breast tomosynthesis; SM, two-dimensional synthetic mammography; MLO, mediolateral oblique; CC, craniocaudal.

The inter-rater reliability (Cohen’s Kappa coefficient) between readers I, and II, was 0.780 (substantial agreement) in FFDM/DBT. Between readers III and IV, the coefficient was 0.773 (substantial agreement) in SM/DBT. For the interpretation methods, the coefficient was 0.745 (substantial agreement) between FFDM/DBT and SM/DBT (***Table 3***).

**Table 3:** Inter-rater reliability with Cohen’s Kappa coefficients.


	COMPARISON	COHEN’S KAPPA COEFFICIENTS

Readers	I and II reading FFDM/DBT	0.780 (substantial agreement)

	III and IV reading SM/DBT	0.773 (substantial agreement)

Interpretation	FFDM/DBT and SM/DBT	0.745 (substantial agreement)


FFDM, full-field digital mammography; DBT, digital breast tomosynthesis; SM, two-dimensional synthetic mammography.

FFDM/DBT and SM/DBT had similar sensitivity (73.1 (38/52) and 71.2% (37/52), *p* = 1.000), specificity (81.3% (109/134) and 82.8% (111/134), *p* = 0.874), and accuracy (79.0% (147/186) and 79.6% (148/186), *p* = 1.00) (***Table 4***).

**Table 4:** FFDM/DBT compared with SM/DBT for the detection of breast cancer.


INTERPRETATION		FISHER’S EXACT TEST	

Sensitivity	FFDM/DBT	38/52 (73.1%)	

	SM/DBT	37/52 (71.2%)	*p* = 1.000

Specificity	FFDM/DBT	109/134 (81.3%)	

	SM/DBT	111/134 (82.8%)	*p* = 0.874

Accuracy	FFDM/DBT	147/186 (79.0%)	

	SM/DBT	148/186 (79.6%)	*p* = 1.000


FFDM, full-field digital mammography; DBT, digital breast tomosynthesis; SM, two-dimensional synthetic mammography.

**Figure 2:**
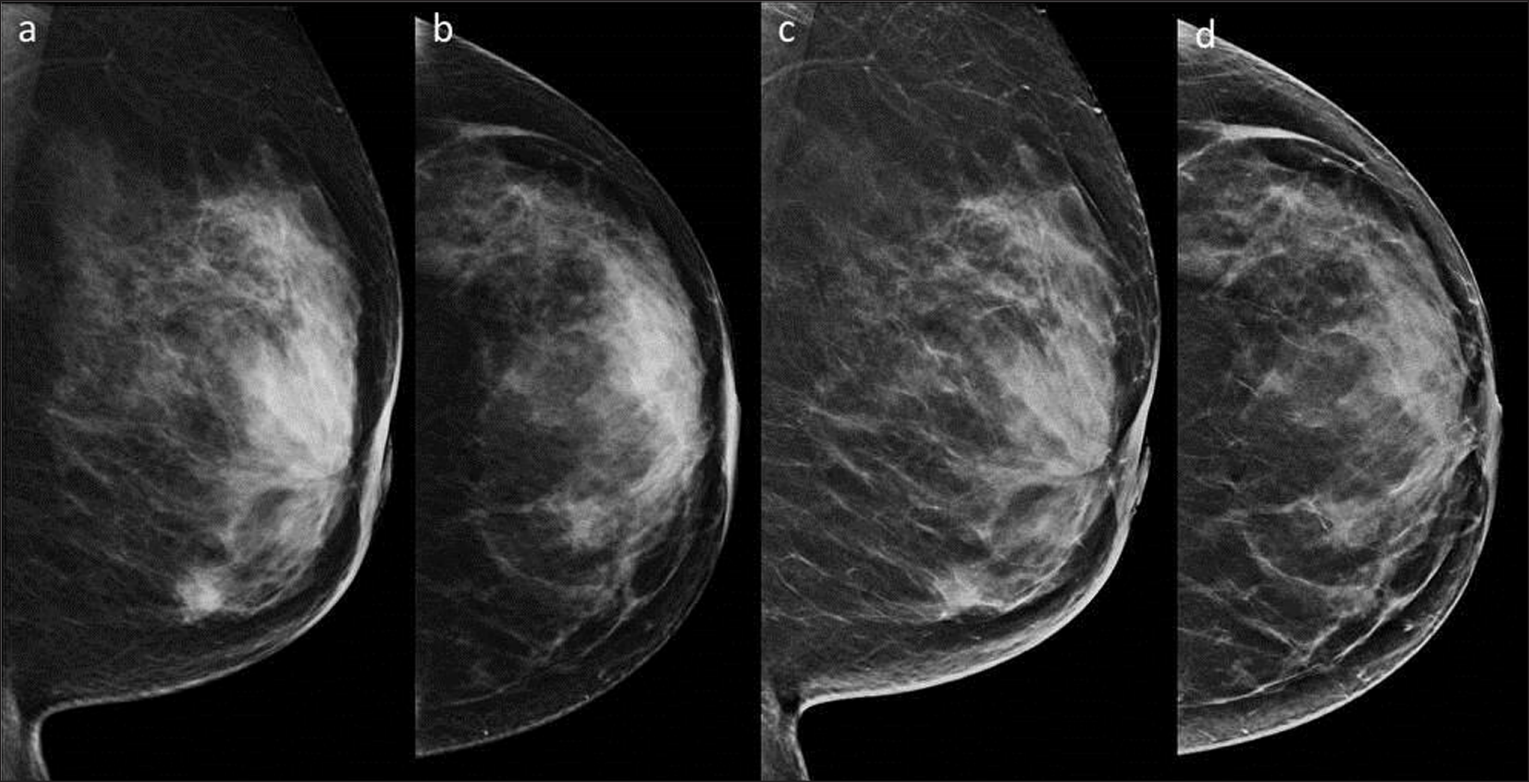
An ill-defined mass was detected with subtle margins in the lower area of the full-field digital mammography (FFDM) mediolateral oblique (MLO) image **(a)** and the inner area of the FFDM craniocaudal (CC) image **(b)**. The mass was rated unclear on the two-dimensional synthetic mammography (SM) MLO **(c)** and CC **(d)** images.

**Figure 3:**
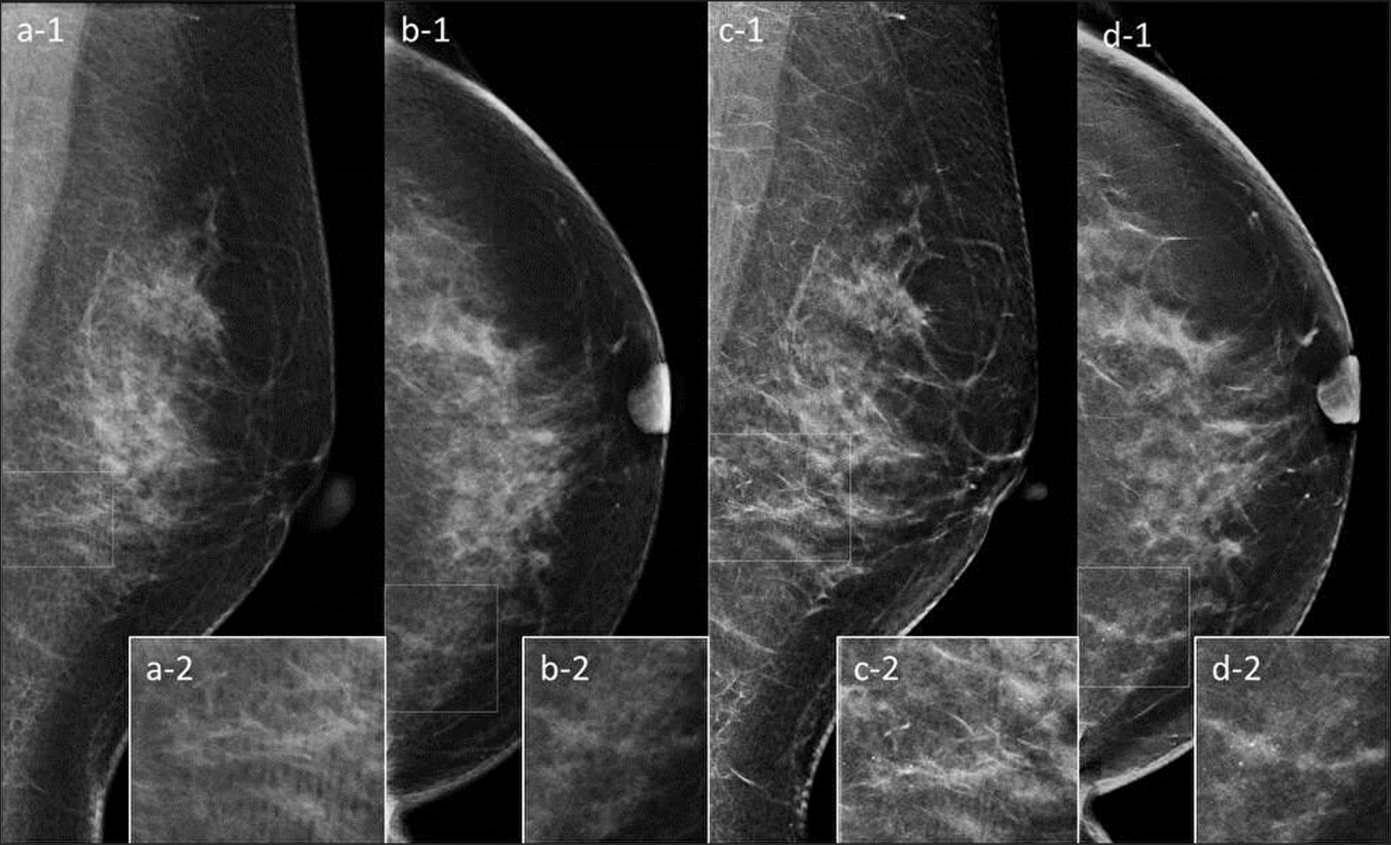
Segmental amorphous microcalcifications were detected in the lower area of the full-field digital mammography (FFDM) mediolateral oblique (MLO) image (**a-1** and **a-2**), and grouped amorphous microcalcifications with distortion were detected in the inner area of the FFDM craniocaudal (CC) image (**b-1** and **b-2**). On the two-dimensional synthetic mammography (SM) images, there were diffuse calcifications in the left breast (**c-1** and **d-1**), which were coarse (**c-2** and **d-2**).

In two IDC cases, abnormalities with subtle margins were compared between FFDM and SM images. One representative case had an ill-defined mass identified by FFDM was unclear on SM (***Figure 2***). Another case had segmental amorphous microcalcifications detected by FFDM whilst on SM, they were interpreted as diffuse coarse calcifications (***Figure 3***).

## Discussion

The present study demonstrated that SM/DBT has a similar performance to FFDM/DBT in the secondary examination of breast cancers. This is consistent with previous studies regarding secondary examination and have shown similar accuracies for FFDM/DBT and SM/DBT [16]. Several studies on breast cancer screening have demonstrated the diagnostic advantage of FFDM/DBT [1,2,3,4,5,6,7,8]. Regarding specific subgroups of women, it has been observed that FFDM/DBT offers a significant improvement in sensitivity in women aged 50–59 years and with breast density of 50% or more, however, this improved sensitivity is not seen with SM/DBT [19]. In the present study, 75.3% of cases were classified as having a dense breast composition. However, the diagnostic accuracy of SM/DBT was not inferior to FFDM/DBT. The two representative cases presented in this study highlight the effectiveness of SM/DBT and demonstrate the limitations of SM. The FFDM images could be used to detect the ill-defined masses from MLO and CC (two-view findings) whereas, the SM image could only show the noticeable density from MLO (one-view finding). This downgraded result is consistent with that of a previous study [20]. Normal structures superimposed on glandular tissue, such as ligaments, can be accentuated [21]; grouped amorphous calcifications on FFDM were interpreted as diffuse coarse calcifications on SM in the present study. These were interpreted as probable malignancies on SM/DBT. Since ill-defined margins of masses delineate the invasive area of breast cancer and amorphous microcalcifications warrant a biopsy for the histological diagnosis, they are important in the detection of abnormalities related to malignancies in clinical judgement. Recently, advancements in SM and DBT have been carried out, including Clarity HD high-resolution 3D^TM^ (70 micron pixel size) for DBT and Intelligent 2D^TM^ (Hologic, Marlborough, MA) for SM [22]. The contrast resolution of the digital image depends on the number of pixel values. Advanced implement is expected to overcome the limitations of SM.

As previously shown [14], the present study demonstrated that SM/DBT required half the radiation dose of FFDM/DBT, similar to the dose required for FFDM alone. Taken together, the present study suggests that DBT complements SM and that SM/DBT produces useful images for secondary examination.

## Conclusion

SM/DBT has a similar performance to FFDM/DBT for secondary examination of breast cancer, while requiring less radiation.
